# Cannabidiol Regulates CD47 Expression and Apoptosis in Jurkat Leukemic Cells Dependent upon VDAC-1 Oligomerization

**DOI:** 10.3390/ph19010095

**Published:** 2026-01-04

**Authors:** Lixing Wang, Suzanne Samarani, Evgenia Fadzeyeva, MariaLuisa Vigano, Alia As’sadiq, Branka Vulesevic, Ali Ahmad, Cecilia T. Costiniuk

**Affiliations:** 1 Faculty of Medicine and Health Sciences, McGill University, Montreal, QC H4A 3J1, Canada; 2 Department of Microbiology and Immunology, McGill University, Montreal, QC H3A 2B4, Canada; 3 Room EM2.3226, Infectious Diseases and Immunity in Global Health Program, Research Institute of the McGill University Health Centre, Montreal, QC H4A 3J1, Canada; 4 Department of Medicine, Division of Clinical and Translational Research, McGill University, Montreal, QC H3A 0G4, Canada; 5 Department of Medicine, Division of Infectious Diseases and Chronic Viral Illnesses Service, McGill University Health Centre, Montreal QC H4A 3J1, Canada

**Keywords:** acute lymphoblastic leukemia, apoptosis, cannabidiol, cannabinoid receptors, CD47, VDAC-1

## Abstract

**Background:** Cannabidiol (CBD) is a major non-psychoactive phytocannabinoid that exerts multiple biological effects in the body. It has been shown to exert anti-cancer effects in a variety of cancer cells, including acute lymphoblastic leukemia of pre-T cell origin (T-ALL), a highly aggressive hematological malignancy. However, the mechanisms underlying CBD’s anti-cancer effects are not fully understood. Furthermore, cancer cells abundantly express surface CD47, which is a negative regulator of phagocytosis and linked with cell survival/death. Little is known about CBD effects on the expression of CD47 in T-ALL cells. The objectives of this study were to address these issues. **Methods:** Studies were conducted in vitro using Jurkat cells and human peripheral blood mononuclear cells in different culture conditions, CBD concentrations, and in the presence or absence of different reagents. **Results:** CBD downregulates CD47 expression and induces apoptosis in Jurkat cells. Similar biological effects of CBD were also observed in primary human CD4^+^ T cells, albeit at reduced levels. The CBD’s effects on CD47 expression and apoptosis were not rescued by a cannabinoid receptor (CBR)-2 agonist, a CBR-2 antagonist, or an anion channel blocker. However, these effects on CD47 expression and apoptosis were significantly rescued by a Voltage-Dependent Anion Channel (VDAC)-1 oligomerization inhibitor. **Conclusions:** Overall, we conclude that CBD downregulates CD47 expression and induces apoptosis involving VDAC-1 oligomerization. Furthermore, they also suggest that CBD’s pro-apoptotic effects on primary human T cells should also be monitored if it is used as an anti-cancer adjuvant or neo-adjuvant therapeutic in cancer patients.

## 1. Introduction

Cannabis has been used by humans for recreational, spiritual, and medicinal purposes for millennia. More than a quarter of cancer patients use it in different forms to relieve pain, anorexia, and anxiety [1]. Divergent and contradictory effects of cannabis usage on cancer per se have been described [2,3,4]. Cannabis contains over 500 active ingredients; more than 125 of them are cannabinoids, terpenophenolic lipophilic compounds with a 21-carbon structural background that exert a variety of biological effects through several canonical and non-canonical cannabinoid receptors in the body [5,6]. As different components in cannabis may exert synergistic or antagonistic effects on cancer in cancer patients, attention is diverting towards using purified cannabinoids for medicinal purposes [7]. Cannabinoids modulate the endocannabinoid system (ECS), which regulates many biological processes and maintains homeostasis in the body. Two major cannabinoids, namely ∆-9 Tetrahydrocannabinol (THC) and Cannabidiol (CBD), isolated from the cannabis plant, have been well characterized. Of the two, THC is psychoactive and addictive and is mainly responsible for the “mood-elevating” effects of cannabis [6]. It is approved for the treatment and management of chemotherapy-induced nausea and pain in cancer patients and for anorexia in AIDS patients [8]. CBD, on the other hand, is devoid of the psychoactive, addictive, and intoxicating effects of THC and is approved for use in some rare forms of epilepsy, opioid use disorder, anxiety, insomnia, and chronic pain. Due to its non-psychoactive effects, CBD is a preferred choice as a therapeutic over THC. It exerts anti-inflammatory, analgesic, anti-epileptic, and anxiolytic effects in the body by interacting with a variety of receptors, such as cannabinoid receptor (CBR)-1, CBR-2, Transient Receptor Potential (TRP) channels, serotonin receptors, and others [5,9]. Interestingly, CBD has been shown to exert anti-cancer effects in different cancer cell types as well as in preclinical studies [9,10,11,12]. It has also been shown to induce apoptosis in leukemic cells through a variety of mechanisms that include the release of Cytochrome c following the elevation of intramitochondrial Ca^++^ [13], CBR-2-mediated upregulation of ROS through upregulating NADPH oxidases [14], and targeting the NOTCH-1 signaling pathway [15]. The phytocannabinoid has also been shown to interact directly with Voltage-Dependent Anion Channel (VDAC)-1 and induce apoptosis in different types of cancer cells [13,16,17]. However, the potential role of CBD-VDAC-1 interaction in CBD-induced death in leukemic cells remains underexplored. Furthermore, little is known about the potential effect of CBD on CD47, a surface glycoprotein that is abundantly expressed in cancer cells and serves as an immune checkpoint (a “do not eat me signal”) [18]. Importantly, it protects cancer cells from death in a cell-autonomous manner when they are exposed to chemotherapy and/or irradiation [19]. Given the intimate linkage of CD47 with cell survival and death, investigating the impact of CBD on CD47 expression is an important research topic. We addressed these issues in this study using the Jurkat cell line, an in vitro cell model for human T-ALL, which is a highly aggressive hematological malignancy with a 5-year survival rate of 44–85% [20,21]. T-ALL accounts for 12–15% of acute lymphoblastic leukemia (ALL) cases in children and approximately 25% in adults. Although current anti-cancer drugs have significantly improved survival in T-ALL patients, there occur relapses, treatment resistance, and toxicities that adversely affect patients’ quality of life, necessitating the search for safer and more effective anti-cancer drugs.

We show here for the first time that CBD downregulates the surface expression of CD47 in Jurkat cells. The phytocannabinoid also induces apoptosis in these lymphoblastic T cells. The CBD-induced downregulation of CD47 expression and apoptosis was significantly rescued by NSC15364, a potent and specific inhibitor of VDAC-1 oligomerization [16].

## 2. Results

### 2.1. CBD Reduces CD47 Expression in Jurkat Cells

**Figure 1:**
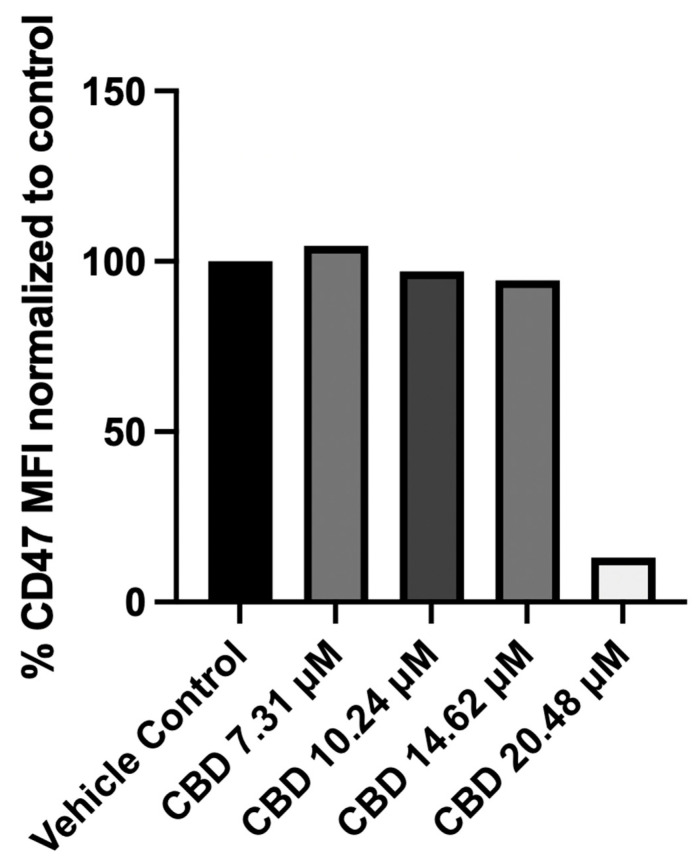
Effect of CBD on CD47 expression in Jurkat cells when cultured in RPMI-1640 containing 10% FBS. After 48 h of exposure, CBD at a 20.48 µM concentration significantly reduced CD47 expression compared with the vehicle control. MFI (normalized with respect to the control) from a typical experiment is shown.

**Figure 2:**
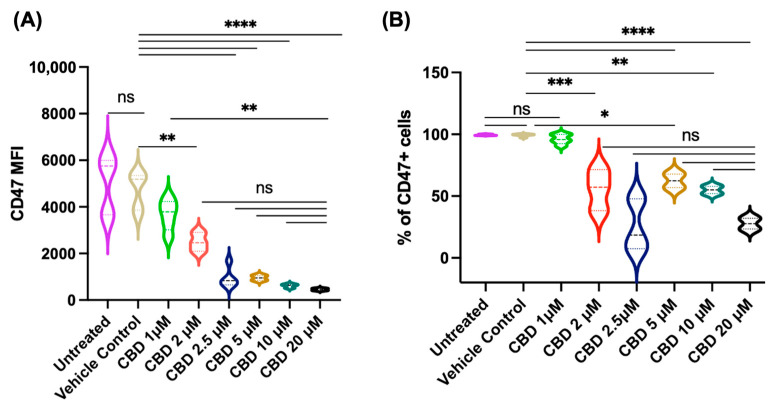
Effect of CBD on CD47 expression in Jurkat cells. Panel (**A**): CBD treatment under serum-free conditions for 24 h downregulates CD47 expression on the cell surface in a dose-dependent manner. Panel (**Β**): The CBD treatment decreases percentages of CD47^+^ cells at >2 μM concentrations of CBD. Statistics: one-way ANOVA with Tukey’s post hoc test. Pairwise comparison labeled as follows: ns: non-significant; *: *p* < 0.05; **: *p* < 0.01; ***: *p* < 0.001; ****: *p* < 0.0001. The same treatment in the two panels is indicated by the same color.

When Jurkat cells were cultured in the culture medium containing 10% FBS for 24 h, the addition of CBD at different micromolar concentrations (2.93–14.62 μM) had little effect on the expression of CD47. At a longer incubation period of 48 h, CBD decreased the expression of CD47 at a higher (20.48 μM) concentration (Figure 1). As several proteins present in serum (e.g., albumin, α-glycoprotein, and lipoproteins) bind CBD (and other cannabinoids) and decrease their availability to cells under in vitro conditions [22,23], experiments were repeated under serum-free conditions. In these experiments, CBD treatment decreased the expression of CD47 in a dose-dependent manner as measured by mean fluorescence intensity (MFI; Figure 2). The percentage of CD47^+^ cells also decreased in a dose-dependent manner when the cells were treated with CBD in serum-free conditions.

### 2.2. CBD Induces Apoptosis in Jurkat Cells

**Figure 3:**
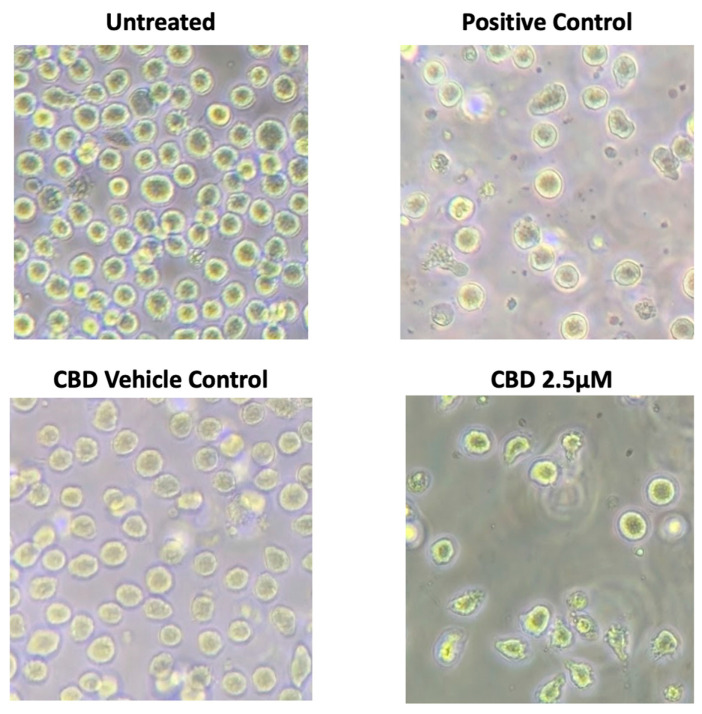
CBD induces apoptotic morphology in Jurkat cells. Photomicrographs (20×) of Jurkat cells following 24 h treatment with 2.5 μΜ CBD. Note marked changes in cell morphology at 2.5 μM compared to vehicle control, similar to cellular morphology seen in the positive control (150 mM ethanol).

**Figure 4:**
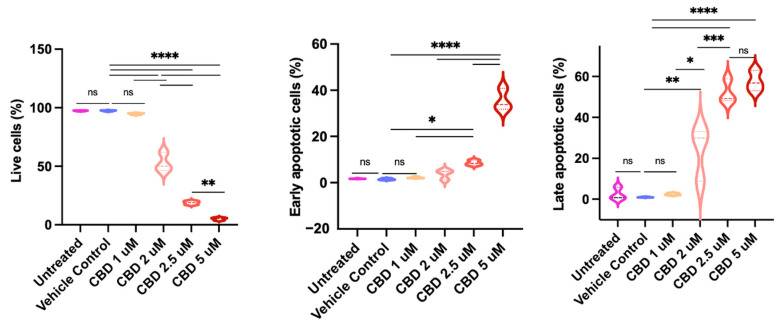
CBD induces dose-dependent apoptosis in Jurkat cells under serum-free conditions. FITC-Annexin V and PI staining after 24 h of CBD treatment. Percentages of live (FITC-AV^−^/PI^−^) cells, early apoptotic (FITC-AV^+^/PI^−^) cells, and late apoptotic (FITC-AV^+^/PI^+^) cells are shown. Statistical comparisons were made by one-way ANOVA with post hoc Tukey tests. The percentage of dead (FITC-AV^−^PI^+^) was constant for all the experimental groups, at around 1%; thus, the data were not shown in the graph. Pairwise comparison labeled as follows: ns: non-significant; *: *p* < 0.05; **: *p* < 0.01; ***: *p* < 0.001; ****: *p* < 0.0001. The same treatment in the three panels is indicated by the same color.

When examined under a light microscope, CBD-treated Jurkat cells exhibited apoptotic morphology (Figure 3). To investigate whether the cells were undergoing apoptosis, they were stained with FITC-Annexin V and PI and examined by flow cytometry. As shown in Figure 4, the percentages of live cells decreased, while those of early apoptotic (FITC-AV^+^PI^−^) and late apoptotic (FITC-AV^+^PI^+^) cells increased in a dose-dependent manner.

### 2.3. CBD Reduces CD47 Expression in Human Primary CD4^+^ T Cells

**Figure 5:**
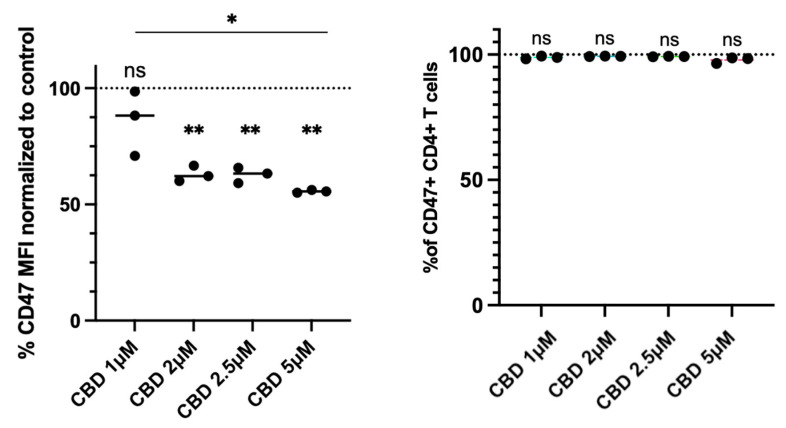
Effect of CBD on CD47 expression in human primary CD4^+^ T cells. CBD reduces the expression of CD47 but not the percentages of CD47-expressing CD4^+^ T cells in human PBMC microcultures. Pairwise comparison labeled as follows: ns: non-significant; *: *p* < 0.05; **: *p* < 0.01.

**Figure 6:**
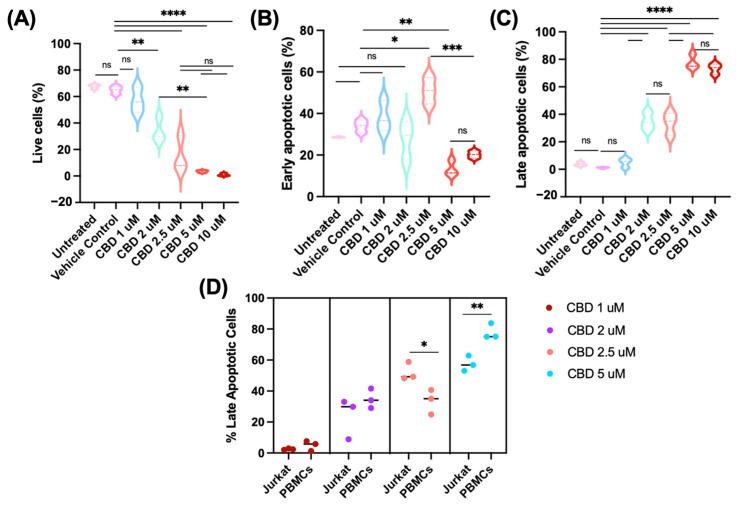
CBD induces dose-dependent apoptosis in CD4^+^ T cells in PBMC microcultures under serum-free conditions. FITC-Annexin V/PI staining was performed after 24 h of CBD treatment. Panel (**A**): percentage of live (FITC-Annexin V^−^/PI^−^) cells, Panel (**B**): early apoptotic (FITC-Annexin V^+^/PI^−^) cells, and Panel (**C**): late apoptotic (Annexin V^+^/PI^+^) cells. The percentages of necrotic (FITC-AV^−^ PI^+^) cells were constant for all the experimental groups (1%) and are not shown. Panel (**D**) compares the percentage of late apoptotic cells at different CBD concentrations for Jurkat and CD4^+^ T cells from PBMC. Pairwise comparison labeled as follows: ns: non-significant; *: *p* < 0.05; **: *p* < 0.01; ***: *p* < 0.001; ****: *p* < 0.0001. The same treatment in the four panels is indicated by the same color.

In view of the CBD-induced reduction in CD47 expression in Jurkat cells, we investigated whether it would also exert this effect on primary human CD4^+^ T cells. For this purpose, we incubated human PBMCs from healthy donors with different concentrations of CBD in serum-free conditions. Results, shown in Figure 5, demonstrate that CBD at >1 μM concentrations reduces the expression of CD47 in primary CD4^+^ T cells gated as CD3^+^CD4^+^ cells among the PBMC (Supplementary Figure S1) by flow cytometry. CD47 expression was downregulated in CD4^+^ T cells, when they were treated with CBD at >1 μM concentrations. However, the amplitude of CD47 reduction was less than that observed in Jurkat cells. Furthermore, percentages of CD4^+^ T cells expressing CD47 were not reduced at any of the CBD concentrations used (Figure 5). In line with these results, CBD treatment decreased the percentages of live cells and increased the percentages of early (FITC-AV^+^PI^−^) and late apoptotic (FITC-AV^+^PI^+^) CD4^+^ T cells (Figure 6). These data demonstrate that CBD concentrations above 2 µM trigger significant cell death in CD4^+^ T lymphocytes under serum-free conditions.

### 2.4. Mechanisms Underlying CBD-Induced Reduction in CD47 Expression

**Figure 7:**
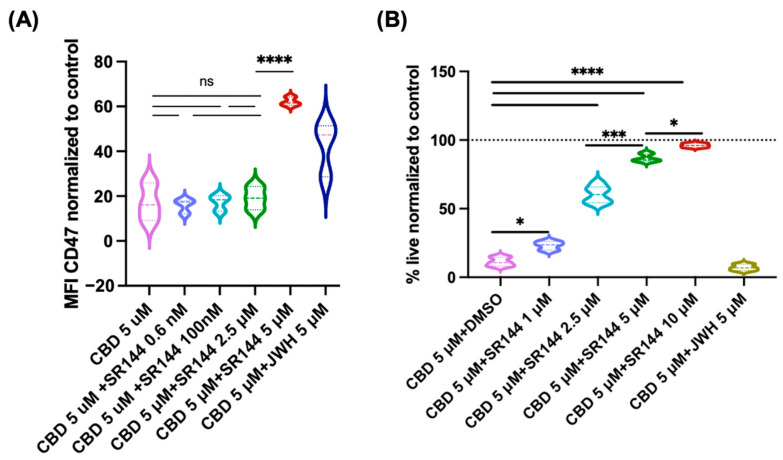
Effect of CB2 receptor agonist (JWH-133) and antagonist (SR144528) on CBD-induced downregulation of CD47 and cell death. The antagonist rescued CD47 expression (Panel (**A**)) and cell death (Panel (**B**)) when used at 5 µM concentrations, whereas the agonist had little effect on these CBD-induced effects in Jurkat cells when used at 5 µM concentrations. Pairwise comparison labeled as follows: ns: non-significant; *: *p* < 0.05; ***: *p* < 0.001; ****: *p* < 0.0001. The same treatment in the two panels is indicated by the same color.

**Figure 8:**
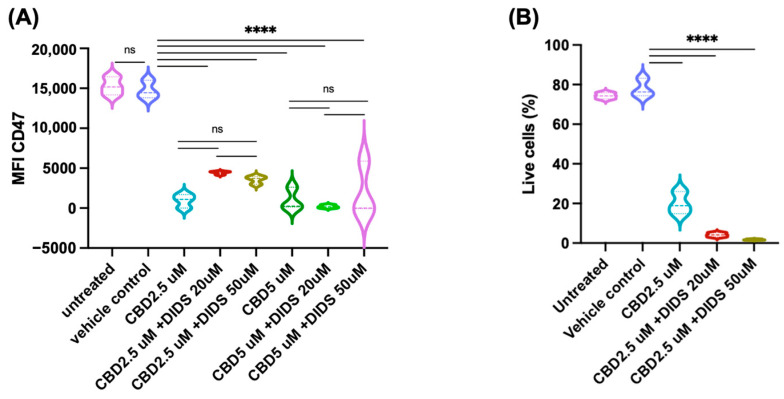
Effects of VDAC blocker DIDS on CBD-induced CD47 down-regulation and cytotoxicity in Jurkat cells. The cells were treated for 24 h with CBD with and without DIDS under serum-free conditions. Panel (**A**): DIDS did not significantly affect CBD-induced downregulation of CD47; Panel (**B**): the inhibitor also did not significantly affect CBD-induced cell death in Jurkat cells at the recommended concentrations. Pairwise comparison labeled as follows: ns: non-significant; ****: *p* < 0.0001. The same treatment in the two panels is indicated by the same color.

**Figure 9:**
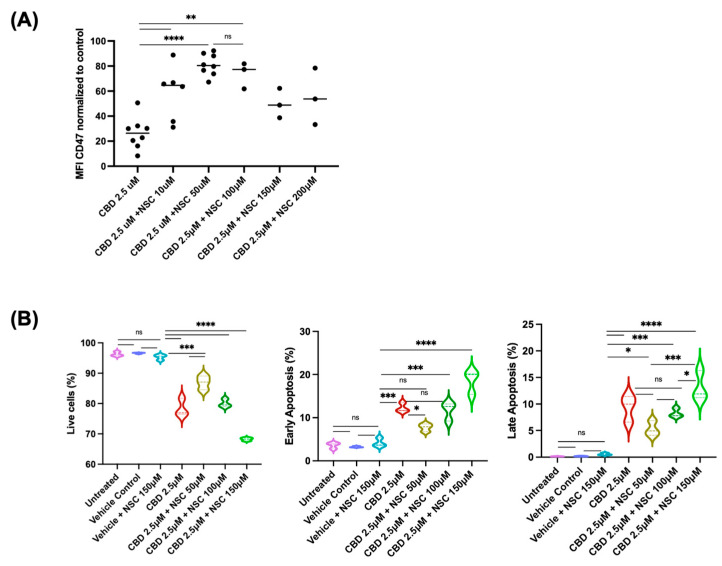
Effects of VDAC oligomerization inhibitor NSC15364 on CBD-induced effects on Jurkat cells. The cells were treated for 24 h under serum-free conditions with CBD (2.5 μM) alone or in combination with the inhibitor. Panel (**A**): The inhibitor rescues CBD-induced CD47 downregulation in Jurkat cells at a 50 μM concentration. The inhibitor’s effect is reduced at higher concentrations. Panel (**B**): The inhibitor reduced CBD-induced apoptosis at a 50 μM concentration; the effect was also reduced at higher concentrations. The results are shown from three independent experiments. Pairwise comparison labeled as follows: ns: non-significant; *: *p* < 0.05; **: *p* < 0.01; ***: *p* < 0.001; ****: *p* < 0.0001. The same treatment in (**B**) panels is indicated by the same color.

To explore the mechanisms through which CBD reduces the expression of CD47 and apoptosis, we investigated the involvement of a selective CB2 receptor agonist (JWH-133) [24], a CB2 receptor antagonist (SR144528) [25], and an inhibitor of anion transporters, DIDS, as well as a VDAC-1 oligomerization inhibitor [26]. The CB2 antagonist SR144528 did not restore CBD-induced CD47 downregulation or rescue CBD-induced apoptosis at nanomolar concentrations (0.6 nM to 100 nM), a dosage suggested for antagonizing CB2 [25]. However, increasing the concentration to 5 μM rescued the CBD-induced reduction in CD47 expression as well as CBD-induced apoptosis in Jurkat cells (Figure 7). Furthermore, the CB2 agonist JWH-133 had little impact on both CBD-induced reduction in CD47 expression and on CBD-induced apoptosis in these cells when used at the recommended concentration (Figure 7). We also investigated the impact of DIDS, an anion channel blocker that also inhibits VDAC-1 oligomerization, an anion channel implicated in CBD-induced cell death [16,27]. Interestingly, the anion channel blocker did not significantly affect CBD-induced reduction in CD47 expression and cell death when used at 20 or 50 μM concentrations (Figure 8). Finally, we investigated the impact of a specific VDAC-1 oligomerization inhibitor, NSC15364 [28], on the CBD-induced effects of CD47 expression and cell viability in Jurkat cells. The oligomerization inhibitor significantly rescued CBD-induced reduction in CD47 expression at 50 μM concentration; however, at higher concentrations (100–200 μM), the inhibitor’s effect was significantly reduced (Figure 9). In line with the effects of NSC15364 on CBD-induced reduction in CD47 expression, the VDAC-1 oligomerization inhibitor also significantly rescued CBD-induced apoptosis in Jurkat cells at a 50 μM concentration, and the rescue effect was lost at higher concentrations (Figure 9). 

## 3. Discussion

CBD and THC are two of the most studied cannabinoids found in the cannabis plant. Due to its non-psychoactive and non-addictive effects, CBD is preferred over THC for medicinal purposes. Currently, it has been approved by the Federal Drug Administration Agency (FDA) and the European Medicines Agency (EMA) for seizures in certain severe and untreatable forms of epilepsy, such as Lennox–Gastaut syndrome, Dravet syndrome, and tuberous sclerosis [29,30]. Furthermore, research has suggested its usage in other human diseases such as cancer, chemotherapy-induced peripheral neuropathy, acne, autism spectrum disorder (ASD), and insomnia [31,32,33,34]. Several in vitro studies have explored the mechanisms by which CBD induces a reduction in cell size and apoptosis in leukemic cells [13,14], breast cancer cells [35], and others [36]. In these studies, a variety of mechanisms have been shown to induce a reduction in cell size and apoptosis in cancer cells. We show here for the first time that CBD treatment leads to a reduction in the surface expression of CD47 as well as apoptosis in Jurkat leukemia cells. We also show for the first time that these CBD-induced effects in this cell line were rescued by NSC15364, a specific VDAC-1 oligomerization inhibitor.

The CBD-induced effects were significantly affected by the presence of serum proteins in the culture medium. Serum-containing culture required at least a ten-fold increase in CBD concentration to induce significant cell death compared to serum-free culture conditions. These results are in line with previous findings in the literature [23]. It has been demonstrated that about 95% of CBD is bound with serum proteins such as albumin and α1-acid glycoprotein and is not available for binding with cancer cells [37,38]. Thus, in vitro studies of CBD on cancer cells pose a conundrum. While the presence of serum masks the cytotoxic effects of CBD, the lack of it in the culture medium renders these cells more prone to CBD’s cytotoxic effects. A caveat, however, is that the results obtained under serum-free conditions may not reflect the real-world physiological situation where albumin is normally present in the tumor microenvironment unless there is extreme cachexia and hypoalbuminemia [39]. As only 5% of the CBD reaching the circulation is available for interaction with cancer and non-cancer cells in the body, this availability may be increased by co-administration of other albumin-binding drugs such as tizoxanide (an active metabolite of the anti-inflammatory and anti-microbial drug nitazoxanide) [37]. Furthermore, CBD is mainly metabolized in the body by the CYP-450 isoenzymes CYP2C19, CYP3A4, and CYP2C9. Co-administration of drugs that induce or inhibit these isoenzymes will affect the bioavailability of CBD in the body, as reviewed in [40].

CD47, also known as the Integrin-Associated Protein, is a ubiquitous and multifunctional glycoprotein expressed on the surface of healthy cells [18,19,41]. It interacts with signal regulatory protein (SIRP)-α, thrombospondin-1, αvβ3 and α2β1 integrins, and Fc receptors. Depending upon the interacting ligand, CD47 regulates phagocytosis, apoptosis, cell migration, immune homeostasis, and cellular metabolism [19,42,43]. It is overexpressed in cancer cells, and the expression is associated with poor clinical outcomes [44]. It acts as a “do not eat me” signal and prevents phagocytosis of CD47^+^ cells by interacting with SIRP-α expressed on the surface of phagocytes [45]. Furthermore, the enhanced expression of CD47 on cancer cells and on immune cells in viral infections may inhibit macrophages and dendritic cells from mounting the host’s innate immune response [46]. Blocking the CD47-SIRPα interactions through monoclonal antibodies or small molecules has been shown to promote cancer regression in preclinical studies. The strategy is actively being investigated in clinical trials with encouraging results [47,48]. In healthy cells as well as in cancer cells, CD47 is clustered in lipid rafts in distinct puncta, whereas its expression becomes decreased and diffused throughout the plasma membrane (unclustered) in apoptotic and aged cells, facilitating their engulfment and removal by phagocytic cells. Furthermore, CD47 may also undergo a conformational change that interacts with SIRPα and promotes phagocytosis [42,49]. To date, this is the first report demonstrating CBD-induced downregulation of CD47 expression in leukemic cells, which, like other cancer cells, overexpress it. A decreased expression of CD47 on cancer cells may make them more susceptible to chemo-, immune-, and radio-therapy. Interestingly, the enzyme glutaminyl-peptide cyclotransferase-like protein (QPCTL) catalyzes the pyroglutamylation and is involved in the maturation of CD47 (and other proteins) and is crucial for the binding between CD47 and SIRPα. QPTCL inhibitors, therefore, prevent maturation of CD47 and its interaction with SIRPα [50]. Further studies are required to investigate the effect, if any, of CBD on the maturation of CD47 through QPCTL. Moreover, CBD-induced downregulation of CD47 in cells of other cancer types should be examined.

Despite extensive research on the anti-cancer effects of CBD, the relative importance of different receptors in CBD-induced apoptosis is still not fully understood. CBD exerts its biological effects through its interaction with various receptors and channels such as Transient Receptor Potential (TRP) channels, Peroxisome-Proliferator-Activated Receptor (PPAR)-γ, VADC-1, G-Protein coupled Receptor (GPR)-55, and others [5,8]. Interestingly, CBD shows little or no affinity for CBR1 and CBR2, which represent canonical cannabinoid receptors. However, CBR1, CBR2, and other receptors (TRPV-1 and PPAR-γ) were shown to be implicated in CBD-induced cell death in different cancer type cells. Although CBD has little or no affinity with CBR1 and CBR2, it acts as a negative allosteric modulator for both receptors [51,52]. Of these two receptors, CBR2 is known to be expressed on immune cells and Jurkat T cells, and CBR2 agonists inhibit T cell proliferation and act as anti-inflammatory and immunosuppressive agents [53]. A previous study by McKallip et al. has shown that the pro-apoptotic effects of CBD in leukemic cells can be blocked by the CBR2 receptor antagonist, SR144528 [14]. However, our study shows that SR144528, as well as a CBR2 agonist, JWH-133, had only non-significant effects on CBD-induced CD47 downregulation and cell death at their recommended sub-micromolar concentrations [25]. It has been demonstrated that at higher concentrations (in the micromolar range), SR144528 exerts off-target effects, including inhibition of the activation of caspases implicated in apoptosis [54]. The previously reported inhibition of CBD-induced cell death by SR144528 may have been caused by these off-target effects [14]. Our results are supported by other studies, which demonstrated that CBD’s apoptotic effects in leukemic cells are not inhibited by blocking CBR1, CBR2, GPR-55, or PPAR-γ receptors [4,13]. CBD was also shown to mediate its pro-apoptotic effects in different cancer cells through interacting with TRP channels [12,55]. Jurkat cells express different types of TRP channels [56]; further studies are needed to determine if any of these channels play a role in CBD-induced apoptosis in these cells.

In the context of CBD-induced cancer cell death, the role of VDAC-1 deserves special attention. CBD has been shown to interact with it directly [13,17], and our results show that a VDAC-1 oligomerization inhibitor, NSC15364, but not DIDS, was able to inhibit CBD-induced cell death in Jurkat cells. Previously, DIDS was shown to inhibit VDAC-1 oligomerization-induced apoptosis in different types of cancer (not leukemia) cells [16,27]. In another study [28], DIDS as well as NSC15364 inhibited ferroptosis, a form of apoptosis induced by oxidative stress and buildup of lipid peroxides. It may be relevant to mention here that DIDS, in addition to inhibiting VDAC-1 oligomerization, also inhibits anion transport channels, ABCA-1, and RAD-51 [57]. Furthermore, we used this reagent to inhibit CBD-induced apoptosis at 20 and 50 µM concentrations, whereas previous studies used it at 70–400 µM concentrations [16,28]. The different doses of DIDS used in our and earlier studies may have yielded different results. Furthermore, different cell types and apoptosis-inducing stimuli may also have contributed to discrepant results.

VDAC-1 is a multifunctional channel located mainly in the outer mitochondrial membrane (OMM), where it acts as a gatekeeper for the transport of ions, nucleotides, and metabolites between mitochondria and cytosol. The channel plays an important role in mitochondrial dynamics, cellular energy production, and the induction of the endogenous/mitochondrial pathway of apoptosis [58]. It also acts as a scaffolding protein and recruits proteins such as hexose kinase (HK)-II and BcL-XL onto the OMM. It transports ATP and other small metabolites across the OMM to HK-II to drive glycolysis, regulate the Kreb’s cycle, and ROS production [58,59]. Cancer cells generally overexpress VDAC-1, which exists in a dynamic balance between monomeric and oligomeric forms [60]. HK-II prevents oligomerization of VDAC-1. CBD permeates through the plasma membrane and is delivered by different fatty acid binding proteins to VDAC-1 on the OMM. CBD-VDAC-1 interaction dislocates HK-II from VDAC-1, induces the latter’s oligomerization, and reduces its conductance for metabolites [61]. The CBD binding with VDAC-1 exposes anionic residues in its inner pore, and the channel becomes permeable to Ca^++^ and restricts flux of metabolites, as visually depicted in the graphic abstract. This leads to the influx of Ca^++^ from the cytosol to the intermembrane space of mitochondria. The buildup of Ca^++^ in this space opens the mitochondrial calcium uniporter (MCU) located in the inner mitochondrial membrane, causing Ca^++^ influx in the matrix, resulting in the loss of mitochondrial transmembrane potential, opening of the mitochondrial permeability transition pore (mPTP), release of Cytochrome C and Apoptosis-Inducing Factor (AIF) from the mitochondria into the cytosol, and triggering the endogenous pathway of apoptosis. Interestingly, the CBD-induced mitochondrial Ca^++^ overload in Jurkat cells precedes the release of Ca^++^ from the ER, which is triggered by the opening of the mPTP and the release of Cytochrome C and AIF from the intermembrane space of mitochondria, and serves as a feedforward mechanism [13]. Thus, CBD directly targets mitochondria through its interaction with VDAC-1, induces its oligomerization, and disturbs calcium homeostasis in acute lymphoblastic leukemia cells [13,27,60]. VDAC-1 oligomerization inhibitors, such as NSC15364, effectively inhibit CBD-induced apoptosis. Interestingly, VDAC-1 oligomerization inhibitors are viewed as promising tools in human diseases involving abnormal cell death, such as Alzheimer’s disease [62].

Our study has certain limitations. The single-cell microculture model used here is inherently incapable of investigating the impact of reduced CD47 expression in CBD-treated cells on their clearance by macrophages. Moreover, the CBD concentrations that reduced CD47 expression and induced apoptosis in our study were 2.5 µM in serum-free and 20.0 µM in serum-replete conditions, respectively. Are these concentrations achieved in the plasma in current CBD dosing regimens? In one study [63], when CBD was administered orally to humans at a 10 mg/Kg dose (on average 700 mg per person) per day for six weeks, peak plasma CBD concentrations ranged from 5.9 to 11.2 ng per ml (equivalent to 0.019 to 0.070 µM concentration). At these plasma concentrations, CBD cannot significantly impact the survival of cancer cells. These observations suggest that for effective anti-cancer treatment, CBD bioavailability would have to be increased by using novel delivery methods. Another limitation worth mentioning is that we did not study how the CBD-induced reduction in CD47 expression may have affected its interactions with CD47 ligands such as thrombospondin-1, αvβ3, and α2β1 that are secreted by or are expressed by Jurkat cells [64,65]. Abrogation of these interactions (in cis or in trans) may impact cellular adhesion, metabolism, and survival. This study also did not explore the mechanism of CBD-induced downregulation of CD47, whether it occurred at the transcriptional, translational, or post-translational level. Finally, we did not carry out all the experiments in the presence of serum in the culture.

Our results show that CBD also induces CD47 downregulation and cell death in primary human CD4^+^ T cells; however, the magnitude of these biological effects is less compared to Jurkat cells. These results are in partial agreement with those of Olivas-Aguirre et al., who showed that CBD does not induce cell death in resting T cells but does so in activated T cells [13]. The discrepancy may also be due to differences in culture conditions: while we incubated T cells in serum-free medium and CD4^+^ T cells were not isolated from PBMC, Olivas-Aguirre et al. purified CD4^+^ T cells and exposed the cells to CBD in the culture medium containing 5% FBS [13]. It may be relevant to mention here that at lower concentrations, CBD has been shown to induce cell proliferation in cancer cells. The effect has been ascribed to the ability of CBD to induce autophagy, which protects cells from stressful conditions such as nutrient deprivation by recycling macromolecules [66,67]. The cannabinoid was also shown to induce apoptosis in murine T cells when used in 1.00 to 8.00 µM concentrations [68]. Overall, these results suggest that at higher (in micromolar) concentrations, CBD may prove immunosuppressive by targeting activated CD4^+^ T cells in the body.

## 4. Materials and Methods

### 4.1. Cell Line and Culture Conditions

Jurkat cells were cultured in the Roswell Park Memorial Institute (RPMI)-1640 medium (Life Technologies, Grand Island, NY, USA) with or without 10% *v*/*v* (indicated in individual experiments) of fetal bovine serum (FBS) and 10 µg/mL streptomycin at 37 °C in a humidified 5% CO_2_ atmosphere. The cells were sub-cultured every 3 days in fresh culture medium.

### 4.2. Cell Treatments and Reagents

Jurkat cells were incubated in 24-well plates (5 × 10^5^ cells per ml) in RPMI-1640 with or without 10% FBS. CBD (Cayman Chemical, Ann Arbor, MI, USA; catalog # 90080) was added to the cell cultures to a final concentration ranging from 6.0 nΜ to 20.5 μM (as indicated in each group). As vehicle controls, cells were treated with equal volumes of methanol. Cells were examined and analyzed following 24- and/or 48 h incubation. In some experiments, cells were co-treated with 4,4′-diisothiocyano-2,2′-stilbenedisulfonic acid (DIDS; Sigma-Aldrich, St. Lois, MO, USA; catalog # D3514) [26]; JWH-133 (Sigma-Aldrich, St. Lois, MO, USA; catalog # SML3627); SR144528 (Cayman Chemical, Ann Arbor, MI, USA; catalog # 9000491) [25]; or NSC15364 (Selleckchem, Houston, TX, USA; catalog # NSC15364) [16]. These reagents were added to the culture medium just prior to the addition of CBD.

### 4.3. Light Microscopy

Jurkat cells, after treatment with different concentrations of CBD in 24-well plate wells, were examined and photographed at 20× magnification using a bright field microscope (Eclipse Ni-E, Nikon, Tokyo, Japan).

### 4.4. Flow Cytometry for CD47 Expression

Following cell treatments, Jurkat cells were washed and treated with a fixable viability dye, Zombie NIR^TM^ (Catalog #423105; Biolegend, San Diego, CA, USA), diluted 1:1000 in phosphate-buffered saline (PBS) to exclude dead cells. Subsequently, Fc receptors were blocked with human Fc Blocking Reagent (Catalog # 130-059-901; Miltenyi Biotec, San Jose, CA, USA), and APC-conjugated mouse anti-human CD47 (Catalog #323123; clone CC2C6; Biolegend, San Diego, CA, USA), diluted 1:100 in FACS buffer (PBS containing 0.5% bovine serum), was used to stain the surface-expressed CD47. After washing with PBS, cells were fixed with 2% paraformaldehyde (PFA) in PBS and analyzed for the expression of CD47 by flow cytometry using BD LSR-Fortessa X20 (B&D). Data acquisitions were performed using FACSDiva v8.0.3 and analyzed by Flow Jo v10.10 (B&D, Mississauga, ON, Canada).

### 4.5. Determination of Apoptosis

Apoptotic cells were detected and enumerated after staining with BD Pharmingen’s Fluorescein Isothiocyanate (FITC)-conjugated Annexin V and Propidium Iodide (PI) kit using flow cytometry. Following cell treatments, Jurkat cells were counted and washed twice. Subsequently, 1.5 μL of FITC-conjugated Annexin V and 1 μL of PI diluted to 50 μL in PBS were used for each sample. This was followed by two washes, and apoptosis was determined by flow cytometry within 30 min post-staining using a BD LSR-Fortessa X20. Data acquisitions were performed using FACSDiva v8.0.3, and analysis was performed by Flow Jo v10.10 (B&D). FITC-Annexin V^+^ and PI^+/−^ cells were considered apoptotic.

### 4.6. Treatments and Staining of CD4^+^ T-Lymphocytes from Peripheral Blood Mononuclear Cells (PBMCs)

Cryopreserved PBMCs from three healthy volunteers were thawed and cultured in RPMI with 10% FBS for 24 h. PBMCs were then washed twice with PBS by centrifuging at 300× *g* at room temperature (RT) for 7 min. Washed cells were transferred into 24-well plate wells (1 × 10^6^ cells per well in 2 mL RPMI-1640) for CBD treatments as described above for Jurkat cells. After the treatments, PMBCs were washed twice and transferred to FACS tubes for staining procedures. PMBCs were first stained with Zombie NIR™ Fixable Viability Kit (Biolegend, San Diego, CA, USA) diluted in PBS (1:1000 by volume) for 15 min at RT in the dark. The cells were then washed twice with FACS buffer and incubated with human Fc Blocking Reagent (catalog # 130-059-901; Meltenyi Biotec, San Jose, CA, USA) diluted in FACS buffer (1:500) for 20 min at room temperature. Finally, PE-conjugated anti-human CD3 (catalog # 12-0038-41, eBioscience/ThermoFisher Scientific, San Diego, CA, USA) and BV650-conjugated anti-human CD4 (catalog # 583737, BD Bioscience, Mississauga, ON, Canada) were added in a master mix in a final staining volume of 100 μL. Samples were incubated for 1 h on ice in the dark and washed 3 times with 300 μL FACS buffer with centrifugation. Washed cells were fixed with 300 μL PFA (2% in PBS) for 15 min in the dark, washed 2 times with 300 μL FACS buffer, and data were collected using a BD LSR Fortessa x20 and analyzed as described above.

### 4.7. Statistical Analysis

One-way ANOVA with Tukey’s post hoc test was used to compare means between different treatments. *p* values < 0.05 were considered significant. The test was performed, and figures were generated using Prism software integrated with Dotmatics v10.4.1.

## 5. Conclusions

In in vitro studies, CBD downregulates CD47 expression and induces apoptosis in Jurkat leukemic T cells. These biological effects were mitigated when the cells were cultured in the medium containing 10% FBS. The CBD-induced downregulation of CD47 and apoptosis were rescued by a VDAC-1 oligomerization inhibitor but not by blocking the CBR2 receptor or by an anion transport inhibitor. CBR-2 agonist (JWH-133) also did not affect the survival of these leukemic cells. Our study supports a model in which CBD-induced oligomerization of VDAC-1 plays a critical role in its pro-apoptotic effects in leukemic cells. The CBD-induced downregulation of CD47 expression and cell death were also seen in primary human CD4^+^ T cells. While CBD may be investigated as an adjuvant therapy for leukemia, its adverse effects on human immune cells must be closely monitored.

## Data Availability

The original contributions presented in this study are included in the article. Further inquiries can be directed to the corresponding author.

## Supplementary Materials

The following supporting information can be downloaded at: https://www.mdpi.com/article/10.3390/ph19010095/s1, Figure S1: The expression of CD47 on primary CD4^+^ T cells.
