# Supplementary material for: Cannabidiol Regulates CD47 Expression and Apoptosis in Jurkat Leukemic Cells Dependent upon VDAC-1 Oligomerization
Source: Pharmaceuticals (Basel). 2026 Jan 4;19(1):95. doi: 10.3390/ph19010095 (PMC12845101; doi:10.3390/ph19010095)
Supplement: Supplementary file 1 [file pharmaceuticals-19-00095-s001.zip › pharmaceuticals-4045239-supplementary.pdf]

Supplementary Figures

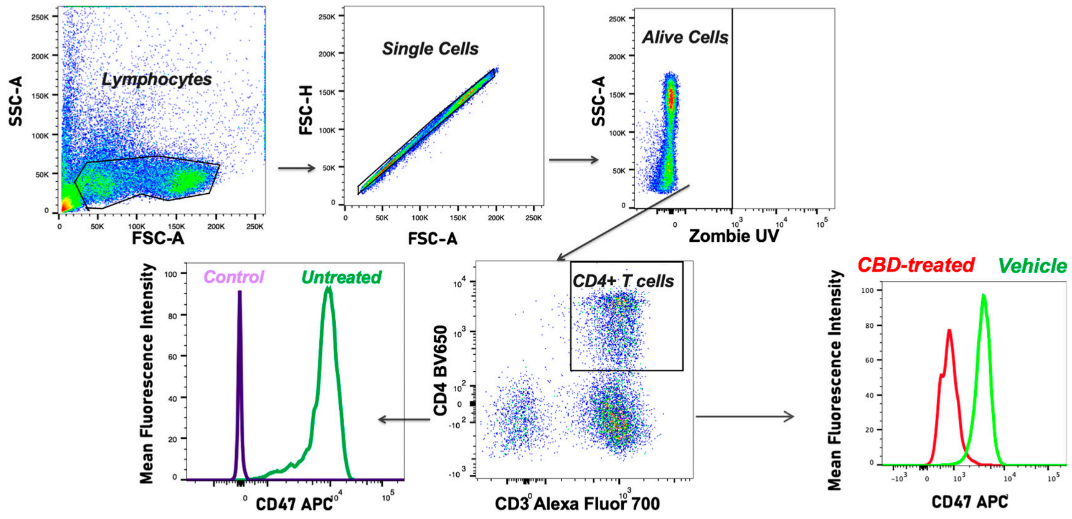

Supplementary Figure 1. The expression of CD47 on primary CD4^{+} T cells. The cell surface expression of CD47 on primary T lymphocytes (green histogram) was measured using flow cytometry and compared to unstained cells (purple histogram). The gating strategies were shown in arrows. The lower right panel compares histograms between CBD-treated and vehicle-treated primary CD4^{+} T cells.
